# A randomised controlled trial of a Mediterranean Dietary Intervention for Adults with Rheumatoid Arthritis (MEDRA): Study protocol

**DOI:** 10.1016/j.conctc.2022.100919

**Published:** 2022-05-03

**Authors:** Tala Raad, Elena George, Anne Griffin, Louise Larkin, Alexander Fraser, Norelee Kennedy, Audrey Tierney

**Affiliations:** a Discipline of Dietetics, School of Allied Health, Faculty of Education and Health Sciences and Health Implementation Science and Technology Cluster, Health Research Institute, University of Limerick, V94 T9PX, Ireland; b Deakin University, Institute for Physical Activity and Nutrition (IPAN), School of Exercise and Nutrition Sciences, Geelong, Victoria, 3220, Australia; c Discipline of Physiotherapy, School of Allied Health, Faculty of Education and Health Sciences, Implementation Science and Technology Centre, Health Research Institute, University of Limerick, V94 T9PX, Ireland; d Department of Rheumatology, University Hospital Limerick, Limerick, V94 T9PX, Ireland; e Graduate Entry Medical School, Faculty of Education and Health Sciences, University of Limerick, Limerick, V94 T9PX, Ireland; f School of Allied Health, Human Services and Sport, Faculty of Science and Engineering, La Trobe University, Melbourne, Vic, 3086, Australia

**Keywords:** Rheumatoid arthritis, Mediterranean diet, Quality of life, Physical function, Randomised trial, Telehealth, Ireland

## Abstract

**Background:**

Rheumatoid arthritis (RA) is the most common type of autoimmune arthritis affecting 0.5–1% of the adult population worldwide. While the primary line of treatment of RA includes pharmacological therapies, people living with the condition often seek non-pharmacological therapies such as diet and exercise in an attempt to attenuate their symptoms. Established, evidence-based dietary guidelines for RA are currently lacking. The MEDRA study aims to explore the effectiveness of implementing, via telehealth, a Mediterranean type diet (MedDiet) compared to a standard healthy diet as per the Healthy Eating Guidelines (HEG) in Ireland in terms of differences in physical function and quality of life in adults with RA living in Ireland.

**Methods:**

The MEDRA study is a parallel, randomised controlled trial delivered through telehealth methods. Forty-four eligible participants who have RA will be randomly allocated to either a MedDiet or HEG group for a 12 weeks intervention period. Primary outcome measures include changes in physical function and quality of life, both of which will be measured using validated questionnaires at baseline, six and twelve weeks. Both intervention arms will attend a total five teleconsultations with a Registered Dietitian (RD). The MedDiet intervention arm focuses on recommendations from the traditional Mediterranean diet and HEG intervention arm will use the dietary recommendations as currently advised in Ireland.

**Discussion:**

This study will provide evidence as to whether dietary treatment of RA can improve physical function and quality of life in a small cohort of participants with RA. The results of the study will be disseminated at national scientific conferences and published in peer-reviewed journals.

**Ethics:**

This protocol has been approved by the Education and Health Sciences Research Ethics Committee at the University of Limerick (2020_09_05_EHS) and by the Health Service Executive Mid-Western Regional Hospital Research Ethics Committee (REC Ref 103/19).

**Trial registration:**

ClinicalTrials.gov NCT04262505. Trial registration date: April 2, 2020.

## 1 Background

Rheumatoid arthritis (RA) is a chronic autoimmune inflammatory disease that primarily affects the joints of the hands and feet, causing malformation and damage to these joints [1]. RA is the most prevalent form of inflammatory polyarthritis affecting 0.5–1% of the global adult population [2]. The condition is three times more common in women than in men with a typical age of onset between 40 and 50 years [3]. RA can affect nearly all organs in the body leading to co-morbid conditions [4]. The prevalence of comorbid conditions reported in different studies varies between 40 and 66% [[5], [6], [7]]. The most commonly reported comorbidities are cardiovascular diseases, gastrointestinal disorders, infections, osteoporosis and depression [8]. The physical function and quality of life of people with RA are severely impacted due to the nature of the condition and the increased risk of morbidities associated with it [9]. Treatment for RA involves lifelong pharmacological adherence to delay the advancement of the disease and ease symptoms [10]. The three general classes of drugs commonly used in the treatment of RA include non-steroidal anti-inflammatory agents, corticosteroids, and disease modifying anti-rheumatic drugs [11]. However, despite all the advancements in the pharmacological treatments, a complete and sustained remission of the condition is largely uncommon among this population [12].

Diet is a major modifiable determinant of chronic diseases and there is a large body of evidence showing that modifications to improve diet quality are directly associated with health benefits [13]. Diet is a topic of interest for people with RA and many have reported that certain foods can help alleviate symptoms and other foods may trigger ‘flare-ups’ leading to swelling, pain and stiffness [14]. There is data confirming positive effects of certain foods such as fish rich in n-3 polyunsaturated fatty acids (PUFAs) and the negative effects of certain unhealthy foods on symptoms of RA [15]. However, the existing evidence on diet and RA remains inconclusive and evidence based dietary guidelines for the management of RA are currently lacking. Recently, the French Society for Rheumatology established nine recommendations on diet for people living with RA which proposes the Mediterranean diet for people with chronic inflammatory rheumatic diseases and more specifically for people with RA mainly due to its beneficial effects on joint symptoms and cardiometabolic health [16].

The traditional Mediterranean diet (MedDiet) is characterised by a high content of vegetables, fruits, legumes, nuts, beans, cereals, grains, fish, and unsaturated fats such as extra virgin olive oil. The MedDiet is regarded as particularly healthy and is promoted for the management of several chronic disease states [17]. The effects of the MedDiet are well researched with many of its health related benefits attributed to its anti-inflammatory properties [18]. Although several case-control studies have suggested that higher consumption of fish, olive oil and cooked vegetables are associated with lower severity of RA [[19], [20], [21], [22], [23]], the evidence on the association between a MedDiet and symptoms of RA is limited. A systematic review including two clinical trials and two prospective studies demonstrated that a Mediterranean diet has beneficial effects in reducing pain and increasing physical function in people with RA [24]. More recently, a meta-analysis found that anti-inflammatory diets including a Mediterranean Diet resulted in notably lower pain compared to habitual diets in people with RA (−9.22 mm; 95% CI −14.15 to −4.29; p = 0.0002; 7 RCTs, 326 participants) [25]. Given these encouraging outcomes, additional studies are needed to explore whether a Mediterranean dietary pattern could improve physical function and quality of life in people with RA.

The telehealth approach for delivering dietary interventions is a safe, convenient and potentially a more inclusive way to capture diverse perspectives compared to face-to-face appointments with participants which is an essential consideration given the recent Covid-19 public health pandemic [26,27]. A systematic review including 23 studies demonstrated that telemedicine could offer a cost-effective and widely accepted approach to deliver consultations remotely in rheumatology [28]. Furthermore, according to Dietitians Australia position statement on telehealth, telephone-delivered dietary consultations are as effective as in-person consultations [29]. The MEDRA study aims to determine, through telehealth methods, the effect of implementing a MedDiet compared to a standard healthy diet as per the Healthy Eating Guidelines (HEG) in Ireland on physical function and quality of life of adults living with RA in Ireland. The specific objectives include (1) assessing the habitual dietary intakes and baseline diet quality of adults living with RA in Ireland; (2) investigating the effects of a MedDiet compared to a standard healthy diet on physical function and quality of life in adults with RA. Using semi-structured focus groups, at the end of the intervention period, the study will also (3) explore the participant experience of taking part in the MEDRA study and identify the self-perceived barriers and enablers towards adhering to the assigned diet.

## 2 Methods

### 2.1 Study design

**Fig. 1:**
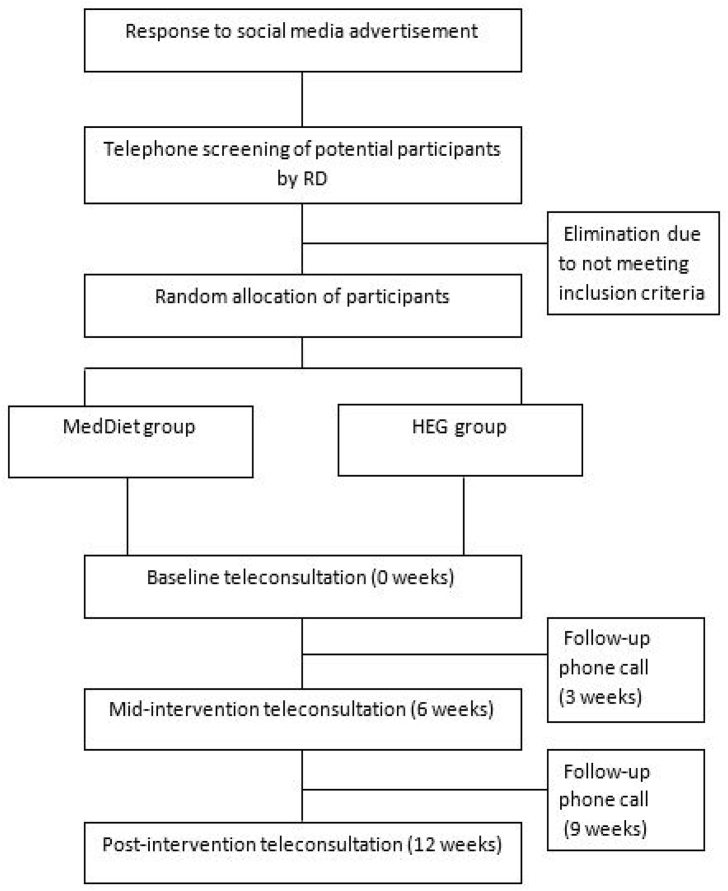
Consort flow chart.

The MEDRA study is a 12-week, parallel group, randomised controlled trial comparing two dietary interventions for the management of RA. This trial is designed in accordance with and adheres to the Consolidated Standards of Reporting Trials (CONSORT) statement [30]. Study participants will be randomly allocated to either a MedDiet group or the HEG group. Participants from both arms will attend five teleconsultations in total with the study Registered Dietitian (RD) over the 12-week intervention period on weeks 0, 3, 6, 9 and 12. For this study, blinding to intervention will not be possible but every effort will be made to manage participants in both groups as equally as possible. The primary endpoint is an improvement in physical function and quality of life of participants over the study period. The study design is detailed in Fig. 1.

### 2.2 Participant recruitment

Participants will be recruited through a collaboration with the patient organisation ‘Arthritis Ireland’ and through their social media platforms (i.e., Facebook, Twitter, Instagram) and from the outpatient rheumatology clinics at University Hospital Limerick (UHL) using poster advertisement.

### 2.3 Participant eligibility

*Inclusion criteria*. Eligible participants will be aged ≥18 years with a definite diagnosis of rheumatoid arthritis according to American College of Rheumatology (ACR)/European League Against Rheumatism (EULAR) criteria [31]. Only English speakers who can complete questionnaires in English and with full access to smartphone or internet will be included in the study.

*Exclusion criteria*. Participants will be excluded if they:

• Refuse or are unable to give informed consent to participate in the study;
• Are women who are currently breastfeeding, pregnant or trying to become pregnant;
• Have commenced nutritional supplements or a new dietary regime in the month prior to study enrolment;
• Are participating in other studies which might affect their diet;
• Have no access to smartphone or Internet

### 2.4 Timeline of assessments

All participants will complete three video teleconsultations meetings through Zoom or Microsoft Teams at baseline, six and twelve weeks. Participants from both groups will also receive two telephone call follow up reviews at weeks three and nine. The video calls will be 30–45 min in duration while telephone call follow-ups will be between 10 and 20 min each. All calls will be delivered by the study RD. All participants will be informed of their diet allocation group during the baseline teleconsultation and will commence the diet the following day or as soon as is feasible for a period of 12 weeks. Participant information including demographic data, smoking status, medication and supplement consumption will be collected at baseline and checked at each video teleconsultation for changes. The baseline teleconsultation will consist of a nutrition and dietetic education session whereby the study RD explains the assigned dietary pattern to participants and how they will be following it over the 12 weeks study period. During the telephone call follow-up reviews, the RD will encourage participants to set three goals that focus on improving diet quality within their assigned diet or maintaining existing positive dietary habits. These goals will be reviewed and modified as needed at follow up sessions. The RD will also monitor participants’ compliance to the assigned diet using a 24-h recall which will be administered over the telephone at weeks three and nine. Table 1 outlines the schedule of measures at each time point.

**Table 1:** Schedule of activities/measures for participants by week of study participation.

Variable	Instrument	Time point				
		Baseline (week 0)	Follow-up (week 3)	Mid-intervention (week 6)	Follow-up (week 9)	Post-intervention (week 12)
Diet intakes	3-day food diary	x		x		x
	24 -hour recall		x		x	
Diet adherence	MEDAS	x		x		x
	Healthy Eating Checklist	x		x		x
Quality of Life	RAQoL Questionnaire	x		x		x
Physical function	HAQ-DI	x		x		x
Anthropometric measures (weight)	patient-report	x		x		x
Anthropometric measures (height)		x				
Physical activity	YPAS	x		x		x
Medications and supplements	patient-report	x				
Smoking status	patient-report	x				
Ethnicity	patient-report	x				

Definition of abbreviations: MEDAS = Mediterranean Diet Adherence Screener; RAQoL = Rheumatoid Arthritis Quality of Life; HAQ DI = Health Assessment Questionnaire- Disability Index; YPAS= Yale Physical Activity Survey.

## 3 Study procedures

### 3.1 Screening assessment and randomisation

Prospective participants who express interest in the study will be contacted by the study RD who will administer a screening questionnaire over the phone. Once deemed eligible, the participant will receive a participant information sheet (PIS) and a consent form to sign and return. Next, participants will be randomly allocated into either a MedDiet group or HEG group using a permuted block randomisation scheme with 1:1 ratio allocation [32]. Randomisation is conducted by a statistician at the University of Limerick, who is not involved in the conduct of the RCT. Next, participants will be invited to complete the online questionnaires. A CONSORT flow chart of participants through the study is provided in Fig. 1.

### 3.2 Dietary interventions

#### 3.2.1 MedDiet group

The MedDiet intervention is based on components of the traditional Mediterranean diet which is primarily plant based and emphasizes intakes of fruits, vegetables and whole grains with the main added dietary fat being extra virgin olive oil (EVOO) [39]. The diet is rich in legumes and raw unsalted nuts, fermented dairy, fish and poultry with very little amounts of red meat [40]. Based on to the MedDiet guidelines and food pyramid, participants in this group will be advised to consume 60–80 ml of EVOO, at least two servings of vegetables and three servings of fruits daily. The study RD will advise participant to include fish at least 3 times a week and a handful of raw unsalted nuts every other day. Participants in the MedDiet group will be encouraged to include red wine in their diet. Red wine will be enjoyed with meals and in moderation (up to 7 glasses per week). Participants will be provided with a copy of a MedDiet guide specifically designed by the study RD to explain the components of the MedDiet. The guide includes the MedDiet food pyramid, 2-week meal plan, recipes, shopping list, ‘no-cooking’ meal options and general tips on how to follow and adhere to the diet. Meal plans and recipes were developed based on the MedDiet guide by Papamiltiadous et al., 2016 [41] with changes to reflect the Irish context.

#### 3.2.2 HEG group

Participants in this group will be advised to adhere to the current Irish Healthy Eating Guidelines [42] with particular emphasis on portion sizes, low fat options and healthy cooking methods. The current Healthy Food for Life – the Healthy Eating Guidelines and Food Pyramid have been developed by the Department of Health in Ireland. The food guide consists of a pyramid representing how different foods and drinks contribute towards a healthy balanced diet. Participants will be advised to limit foods that are high in fat, sugar, and salt to no more than once or twice a week. The diet also emphasizes the consumption of 5–7 portions of fruits and vegetables per day. All participants will be provided with resources to inform them of these guidelines and sample meal plans that are readily available on the Healthy Ireland website (HSE, http://www.hse.ie). A copy of the 101 Square Meals book from Safefood Ireland will be provided to participants in this group. The book includes shopping tips, food safety messages, menu planning advice as well as some healthy treats and snacks ideas. The book uses the Healthy Food for Life Guidelines to help individuals plan healthier meals and will help participants to have a varied and healthy diet while getting the best value for their money.

The diet will be modified and tailored to the participant's preferences by the study RD. This is first achieved based on the assigned diet and overarching principles, however within this, the RD works with the participants on a personal level to ensure the dietary recommendations were achieved. For example, if a person didn't like a recommended food, an alternative of equal nutritional value will be recommended based on participants' preferences. The RD will also manage participants who have allergies to specific food and food components. In the case of food allergies, the RD will evaluate and ensure the nutritional adequacy of an allergen-free diet and suggest appropriate substitutions or individual food replacements.

### 3.3 Focus groups

Semi-structured focus groups from each intervention arm will be conducted at the end of study period. The focus groups will aim investigate participants’ experiences in the MEDRA study and explore the self-perceived enablers and barriers towards adhering to the dietary intervention. A moderator who is a member of the research team but not involved directly with the participants will facilitate the semi-structured focus groups of approximately 4–5 people in an open discussion. Open-ended questions will be asked to encourage the participants to express their views openly and spontaneously. Each focus group will last around 50–60 min and will be audio recorded.

## 4 Outcome measures

The outcome measures and their corresponding timelines are presented in Table 1. Primary outcome measures include physical function and quality of life. Patient information including demographic data, smoking status, medication and supplement consumption are collected at baseline and any changes or additions in pharmacological treatment will be noted during the study period.

### 4.1 Physical function and quality of life

The Rheumatoid Arthritis Quality of Life instrument (RAQoL) is the first patient-completed instrument specifically designed for use with people with RA. It entails 30 questions and respondents are required to indicate whether each of the questions applies to them. Scores range from 0 to 30, with a high score representing poor quality of life [33]. The Health Assessment Questionnaire – Disability Index (HAQ-DI) will monitor changes in the physical function of participants [34]. It is a self-administered questionnaire with 20 questions designed to estimate a patient's level of upper and lower extremity functioning. The total HAQ-DI score is calculated by summing the highest score in each of the 8 domains and dividing the sum by 8, yielding a score ranging from 0 (no disability) to 3 (severe disability). Participants will be required to complete the RAQoL and HAQ-DI questionnaires at baseline, six and twelve weeks.

### 4.2 Diet and physical activity

Participants will be expected to complete a 3-day food diary to include 2 weekdays and 1 weekend day at three time points (baseline, six and twelve weeks) using the Libro - Food Diary & Nutritional Analysis mobile application from Nutritics [35]. The Libro mobile application will be used for logging foods and will help participants keep track of their food and physical activity. Once consent is obtained, all participants will receive a link via email to download the Libro mobile application on their mobile phones. Participants will also receive a link to access the beginner's guide to using Libro which will help them navigate through the application and learn how to add foods and exercise to their diet logs. Any challenges faced by participants will be resolved with the study RD. The 3-day food diary will be used to assess habitual diet and dietary compliance. Participants will be asked to record their intakes while using plate size measurements throughout the day to avoid recall errors. During every video consultation at baseline, six and twelve weeks, the RD will examine the food diaries to check for any missing detail or errors so that they can be amended in consultation with the participant. To check the accuracy of portion size, household measures will be used. Data from food diaries will be exported to Nutritics Diet Analysis software for analysis at baseline, six and twelve weeks. Compliance to the Mediterranean diet will be assessed using the MEDAS from the PREDIMED study [36]. Given the lack of a validated checklist that can accurately assess the adherence towards adherence to the Healthy Eating Guidelines, the authors developed an 11-item checklist based on the dietary guidelines to measure the adherence of participants in the HEG group. The Yale Physical Activity Survey (YPAS) will monitor changes in physical activity [37]. The YPAS includes questions about low intensity, high and leisure activities. The summary indices from YPAS i.e. total activity time and energy expenditure will be used to rank participants based on their activity levels (e.g. Low, moderate, high).

### 4.3 Anthropometric measures

Anthropometric data collection will include measurement of participant's height (cm) and weight (kg). Protocol for the measurement of height and weight will be developed based on the measurement techniques within the International Standards for Anthropometric Assessment published by the International Society for the Advancement of Kinanthopometry [38] and will be provided to the participants prior to the baseline teleconsultation. Participants will be asked to report their height (recorded to the nearest 0.1 cm) during baseline teleconsultation. They will be asked to measure their height using a portable height board and to remove any footwear or headgear while measuring their height. Weight (recorded to the nearest 0.1 kg) will be reported at three time points, baseline, six and twelve weeks. Participants will be advised to use the same weighing scales for the duration of the study and to weigh themselves at the same time of the day (in the morning preferably) and with minimal clothing. All participants are advised to repeat the process three times and to calculate the average to ensure accurate measurements. Whenever possible, anthropometric measurements must be performed in the presence of another person able to assist.

## 5 Data management

The study RD will be responsible for the secure storage of participant data. Study data will be pseudo-anonymized and archived in a password-protected database. At each data collection time point, data are directly entered by the RD into an online cloud-based clinical data platform (Castor Electronic Data Capture (EDC)) hosted on secure servers. Study data will only be accessible to the coordinating and principal investigators.

## 6 Sample size

The sample size calculation was based on the primary outcome measure. To detect a difference of 0.68 units (clinically relevant) in HAQ-DI [43] with 80% power and α = 0.05, 40 participants are needed in total. Adjusting for a potential 10% dropout, the sample size was determined to be 44 participants.

## 7 Statistical analysis

Data will be transferred from Castor EDC into a Microsoft excel spreadsheet, cleaned and imported into SPSS for coding and analysis. Primary outcomes will be analysed based on an ‘intention-to-treat’ principle. Descriptive statistics (M, SD, N and %) will be used to explore demographic and personal characteristics and dietary intakes of participants. Independent samples t-tests and chi-square tests will be used, as appropriate, to investigate any differences between males and females, and to examine diet adherence scores at baseline. Independent t-tests and non-parametric alternatives will be used to assess differences between the two diet groups. The threshold for statistical significance will be set to p = 0.05.; SPSS version 25 will be used for statistical analyses. Differences in outcomes for the two groups will be compared while adjusting for potential confounders such as age, medications and other lifestyle factors.

The focus groups will be audio-recorded and transcribed verbatim. According to Braun and Clarke (2013), data analysis method comprises seven steps: transcription, reading, coding, searching for themes, reviewing themes, defining themes, and finalizing [44]. The transcripts will first be read and coded by two independent investigators. Codes will be compared and discussed to reach consensus. Thematic analysis will be used to inductively code themes that appear significant. Defined themes will then be grouped into categories, as either a perceived barrier or enabler. A second analysis will follow to compare between themes across the two different intervention groups. Analysis of data will mostly be descriptive.

## 8 Ethics and dissemination

Approval to carry out the study was obtained from the Health and Safety Executive (HSE) Mid-Western Regional Hospital Research Ethics Committee (REC Ref 103/19) and from the Education and Health Sciences Research Ethics Committee at the University of Limerick (2020_09_05_EHS). The study will be conducted in accordance with the Good Clinical Practice (GCP) guidelines and written informed consent will be obtained from all study participants. The consent form will contain all the information participants will need to know about participating in the study. They will also be asked if they wish to be involved in the focus groups at the end of the intervention period which are an optional part of the study. There are no additional incentives provided for participation in this trial.

Dissemination of study findings will take place through publications, seminars, blogs and written relevant information to patient organisations. Individual results will be made available to participants upon their request to the study RD.

## 9 Discussion

Despite remarkable advancements in the pharmacological treatments of RA, other management approaches are commonly sought by individuals living with the condition [11,45]. There is a small number of randomised controlled trials and very little evidence on diet and RA. Having a better understanding of the impact of dietary interventions, as an approach for the management of RA will lead to development of dietary guidelines for this population. The MedDiet has been gaining popularity not only in the scientific literature, but also in clinical settings due to its many health benefits. While there is evidence to suggest that this dietary pattern is beneficial for inflammatory conditions, the evidence to recommend this dietary pattern for people with RA is still lacking. The proposed study aims to assess the effect of implementing a MedDiet among adults with RA in Ireland and to determine whether the MedDiet is superior to a standard healthy diet.

The MEDRA study has some limitations that need to be acknowledged. The intervention period is 12 weeks, this is a somewhat a short duration for a dietary intervention compared to other dietary interventions [46]. The sample size is small, and recruitment of study participants is conducted mainly through social media platforms so participants in the MEDRA study may represent a selected group of people with RA and potential selection bias must be evaluated. Further, while both primary outcome measures are clinically relevant, they are subjective measures and must be considered when interpreting results. Moreover, the fact that the MEDRA study is an unblinded study may introduce additional bias. The strength of this research is in its randomised controlled trial design. A trained RD is responsible for delivering both dietary interventions to ensure consistent high quality patient support. Focus group discussions at the end of the intervention will facilitate a deeper exploration into the enablers and barriers of the dietary interventions which is crucial for designing future interventions. Furthermore, the MEDRA study is carried out in collaboration with a consultant rheumatologist and, therefore, results may easily be shared with treating physicians in Ireland.

Most of the studies evaluating the efficacy of the MedDiet were conducted in Mediterranean populations [47]. As such, it is necessary to explore whether such an intervention is effective and in non- Mediterranean countries and among non-Mediterranean populations. To our best knowledge, the MEDRA study is the first randomized controlled trials assessing the effect of a MedDiet in an RA population in Ireland. The MEDRA study will provide evidence as to whether a dietary intervention can improve physical function and quality of life in adults living with RA. Given that the key elements that constitute the MedDiet such as fish, fresh fruits and vegetables, legumes, whole grains and extra virgin olive oil are all readily available and accessible in Ireland, the study will provide insights into the possibility of incorporating these components in an Irish context for people with RA. This, in turn, infer the potential translation to other inflammatory conditions.

## Ethics approval and consent to participate

The MEDRA study has been approved by the Education and Health Sciences Research Ethics Committee at the University of Limerick (2020_09_05_EHS) and by the Health Service Executive Mid-Western Regional Hospital Research Ethics Committee (REC Ref 103/19).

Informed written consent is obtained by all participants prior to commencing the study.

## Data statement

Data sharing is not applicable to this article as no datasets were generated or analysed during the current study.

## Funding

The study is supported by the School of Allied of Allied Health at the University of Limerick.

## Declaration of competing interest

The authors declare that they have no known competing financial interests or personal relationships that could have appeared to influence the work reported in this paper.
